# Estimating influenza-associated mortality in New Zealand from 1990 to 2008

**DOI:** 10.1111/irv.12292

**Published:** 2014-10-24

**Authors:** Tara Kessaram, James Stanley, Michael G Baker

**Affiliations:** a University of OtagoWellington, New Zealand; b Dean's Department, University of OtagoWellington, New Zealand; c Department of Public Health, University of OtagoWellington, New Zealand

**Keywords:** Mortality, influenza, statistical regression

## Abstract

This study used Poisson regression modelling to estimate influenza-associated mortality in New Zealand for 1990–2008. Inputs were weekly numbers of deaths and influenza and RSV isolates. Seasonal influenza was associated with an average of 401 medical deaths annually from 1990 to 2008, a rate of 10·6 (95% CI: 7·9, 13·3) per 100 000 persons per year, which is 17 times higher than recorded influenza deaths. The majority (86%) of deaths occurred in those 65 years and over. There was no clear decline in influenza-associated mortality in this age group over the course of the study period.

## Background

Standard mortality statistics markedly under-estimate influenza-associated deaths. Diagnosis of influenza's role in precipitating important secondary events (e.g. myocardial infarctions) can be difficult.^1^ This leads to under-diagnosis of influenza as a contributor to morbidity and mortality. Such under-ascertainment is compounded by reporting practices for cause of death, which favour recording chronic diseases as the underlying cause.^2^

Several statistical modelling techniques have been developed to overcome the challenges above (see Thompson *et al*^3^ for a review). Poisson regression models (used in this study) incorporate virological data on the circulation of influenza [and other viruses, such as respiratory syncytial virus (RSV)], and can also account for excess mortality beyond seasonal variation.

New Zealand's only previous influenza mortality estimate was based on Poisson modelling for 1980–1992, which produced an estimated average of over 400 influenza-associated deaths per year.^4^,^5^ New Zealand requires updated information to better inform influenza prevention strategies and to supply a southern hemisphere perspective on the epidemiology and impact of influenza.

## Methods

### Data sources

Weekly mortality counts for New Zealand from 1990 to 2008 were obtained from the Mortality Collection (a comprehensive data set maintained by the Ministry of Health). Underlying cause of death (based on ICD code) was used to classify deaths into two groups treated independently for subsequent analysis: respiratory and circulatory deaths (ICD-9 390-519; ICD-10 I00-I99 and J00-J99); and all cause medical deaths (ICD-9 000-629, 680-739, 780-799; ICD-10 A00-N99, R00-R099; excluding mortality associated with pregnancy/childbirth, perinatal conditions and injuries.)

Population data from the New Zealand Census of Population and Dwellings (Statistics New Zealand) in 1991, 1996, 2001 and 2006 gave NZ population counts for every week in the study period (using linear interpolation and extrapolation for the more recent period). The log of these counts was used as an offset variable in the Poisson regression models.

Weekly counts of influenza A, influenza B and RSV in New Zealand were obtained from the Weekly Virology Reports [Environmental Science and Research (ESR)]. These counts combine data on positive isolates from (i) influenza sentinel surveillance in general practices (collected May to September each year) and (ii) regional laboratories (mostly hospital patients, covering the entire year). RSV isolates are identified by ESR and hospital laboratories from specimens received throughout the year.

### Model

For each cause of death category (respiratory and circulatory; all medical) and age grouping (all ages; under 65; 65 plus), we created independent Poisson regression equations that provided the best fit of the observed pattern of mortality for the period 1990–2008. All regression models were fitted using Proc Genmod in sas 9.1 (SAS Institute, Cary, NC, USA).

Our model is based on that of Thompson *et al*^6^ with the predicted number of deaths per week estimated as:


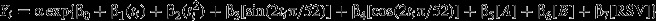


where subscript *i* is week number; *Y*_i_ the output of the modelling process for that week (predicted mortality count); α is the population offset (accounts for population size changes over time); and *t*_i_ represents number of weeks since the start of the modelling period. For the estimated regression parameters, *β*_0_ is the intercept (number of deaths predicted at *t*_0_, in the hypothetical absence of any influenza virus); *β*_1_(*t*_i_) models linear trend and *β*_2_(*t*_i_^2^) models quadratic trend in mortality over time. Seasonal fluctuations in deaths are modelled with the cyclical parameters *β*_3_[sin(2*t*_i_π/52)] and *β*_4_[cos(2_t_iπ/52)]. The final three parameters represent virus counts in the population: *β*_5_[A] and *β*_6_[B] are the weekly influenza A and B counts, respectively; and *β*_7_[RSV] is the weekly RSV count. For the modelling, we assumed a Poisson distribution of model error terms and used a log-link for the relationship between the covariates and the mortality count data.^6^

From this fitted equation, we calculated for each week the number of predicted deaths, when first the influenza A term and then the influenza B term were set to zero. This provided the number of deaths expected in the absence of both influenza A and B. These weekly totals were summed to provide yearly estimates of influenza-associated deaths.

Mean number of influenza-associated deaths was calculated across all study year estimates, with 95% confidence intervals for the overall estimate calculated using the standard error of these mean counts (t-distribution) – results are expressed as mortality rates per 100 000 population. Confidence intervals for annual rates (Tables 2, 3) were calculated on a log scale, using the yearly sum of excess deaths as a Poisson variable (p. 80).^7^

## Results

**Figure 1:**
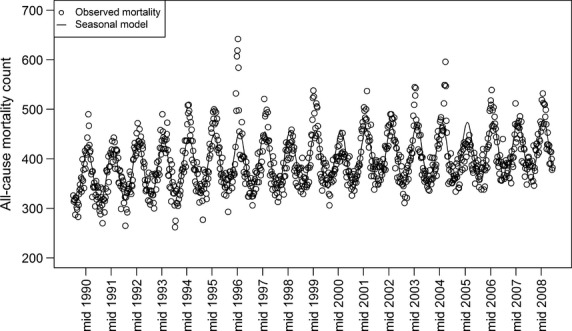
Seasonal pattern (and observed counts) for all medical cause mortality for ages 65 years and older, 1990–2008.

Table 1 summarises the yearly counts of influenza A, influenza B and RSV isolates used in the regression model, along with the observed number of deaths due to respiratory and circulatory causes, and all medical causes. Figure 1 presents the seasonal mortality component of the model for those aged 65 plus (seasonal pattern as solid line; weekly observed all-cause medical mortality as circles).

**Table 1:** Yearly counts of influenza A, influenza B and RSV isolates in New Zealand (source: ESR Weekly Virology Reports) and observed number of deaths for respiratory and circulatory causes, and all medical causes (source: Ministry of Health Mortality Collection) between 1990 and 2008, New Zealand

				Respiratory and Circulatory Mortality			All Medical Causes Mortality		
Year	No. influenza A isolates	No. influenza B Isolates	No. RSV isolates	Under 65	65 and over	All ages	Under 65	65 and over	All ages
1990	322	36	580	2255	11 909	14 164	5551	18 684	24 235
1991	13	145	477	2210	12 077	14 287	5424	18 889	24 313
1992	286	46	514	2273	12 608	14 881	5391	19 693	25 084
1993	285	152	556	2116	12 542	14 658	5279	19 817	25 096
1994	457	6	652	1998	12 404	14 402	5095	20 020	25 115
1995	165	385	502	2021	12 708	14 729	5250	20 505	25 755
1996	766	5	816	2010	12 928	14 938	5255	20 893	26 148
1997	373	377	643	1908	12 394	14 302	5078	20 373	25 451
1998	488	2	789	1762	11 429	13 191	4959	19 851	24 810
1999	664	146	911	1834	12 581	14 415	5135	21 098	26 233
2000	187	66	840	1725	11 297	13 022	4856	20 071	24 927
2001	412	242	566	1728	12 083	13 811	4908	21 250	26 158
2002	534	170	816	1679	12 084	13 763	4869	21 422	26 291
2003	1039	1	795	1624	11 752	13 376	4861	21 055	25 916
2004	829	89	632	1623	12 161	13 784	4906	21 702	26 608
2005	111	730	724	1590	11 123	12 713	4859	20 478	25 337
2006	756	6	686	1557	11 727	13 284	4867	21 628	26 495
2007	588	156	659	1584	11 148	12 732	4806	21 399	26 205
2008	420	622	707	1533	11 223	12 756	4734	21 572	26 306

Based on respiratory and circulatory mortality, there were on average 305·8 influenza-associated deaths per year (95% CI: 235·2, 376·3). This is a rate of 8·1 deaths per 100 000 persons (95% CI: 6·3, 9·9 per 100 000) (Table 2). Mortality varied by study year and age group with 89·4% of deaths in those 65 plus years of age. For all medical cause mortality (Table 3), there were on average 400·5 influenza-associated deaths per year (95% CI: 294·3, 506·6). This is a rate of 10·6 deaths per 100 000 persons (95% CI: 7·9, 13·3). Mortality varied by study year and age group with 86·2% of deaths in those 65 plus years of age.

**Table 2:** Poisson regression model estimates of influenza-associated mortality rates per 100 000 persons based on respiratory and circulatory data (1990–2008); mean mortality rates with 95% confidence intervals

Mortality rates per 100 000 person years (95% confidence intervals)						
	Under 65		65 and over		All ages	
Year	Rate	95% CI	Rate	95% CI	Rate	95% CI
1990	0·8	(0·5, 1·2)	49·0	(42·4, 56·7)	6·2	(5·4, 7·2)
1991	0·3	(0·2, 0·6)	8·2	(5·8, 11·7)	1·2	(0·9, 1·6)
1992	0·6	(0·4, 1·0)	39·1	(33·4, 45·8)	5·1	(4·4, 5·9)
1993	0·8	(0·6, 1·2)	43·9	(37·8, 50·9)	5·8	(5·0, 6·6)
1994	0·9	(0·6, 1·2)	63·5	(56·2, 71·7)	8·1	(7·2, 9·1)
1995	1·0	(0·7, 1·4)	37·2	(31·8, 43·6)	5·2	(4·5, 6·0)
1996	1·3	(1·0, 1·8)	105·5	(96·2, 115·8)	13·6	(12·4, 14·9)
1997	1·2	(0·9, 1·7)	62·1	(55·1, 70·0)	8·4	(7·5, 9·4)
1998	0·8	(0·5, 1·1)	61·4	(54·4, 69·2)	8·0	(7·1, 8·9)
1999	1·2	(0·9, 1·7)	85·9	(77·7, 95·0)	11·3	(10·3, 12·5)
2000	0·4	(0·2, 0·6)	23·1	(19·0, 28·0)	3·1	(2·6, 3·7)
2001	0·9	(0·7, 1·3)	55·9	(49·4, 63·3)	7·6	(6·7, 8·5)
2002	1·0	(0·7, 1·4)	65·1	(58·1, 72·9)	8·7	(7·8, 9·7)
2003	1·5	(1·1, 2·0)	125·3	(115·6, 135·8)	16·5	(15·3, 17·9)
2004	1·2	(0·9, 1·7)	95·3	(86·9, 104·5)	12·7	(11·7, 13·9)
2005	1·1	(0·8, 1·5)	34·7	(29·8, 40·3)	5·2	(4·6, 6·0)
2006	1·0	(0·7, 1·3)	80·3	(72·8, 88·6)	10·7	(9·8, 11·8)
2007	0·9	(0·6, 1·3)	64·1	(57·5, 71·5)	8·7	(7·9, 9·7)
2008	1·2	(0·9, 1·6)	58·9	(52·6, 65·9)	8·4	(7·6, 9·3)
Mean (1990–2008)	1·0	(0·8, 1·1)	61·0	(47·2, 74·8)	8·1	(6·3, 9·9)

**Table 3:** Poisson regression model estimates of influenza-associated mortality rates per 100 000 persons based on all medical cause data (1990–2008); mean mortality rates with 95% confidence intervals

Mortality rates per 100 000 person years (95% confidence intervals)						
	Under 65		65 and over		All ages	
Year	Rate	95% CI	Rate	95% CI	Rate	95% CI
1990	1·2	(0·8, 1·6)	57·2	(50·0, 65·4)	7·6	(6·7, 8·6)
1991	0·4	(0·2, 0·7)	5·4	(3·5, 8·4)	0·9	(0·7, 1·3)
1992	1·0	(0·7, 1·4)	47·2	(40·9, 54·6)	6·4	(5·6, 7·3)
1993	1·2	(0·9, 1·7)	50·0	(43·5, 57·5)	6·9	(6·1, 7·8)
1994	1·4	(1·1, 1·9)	78·4	(70·2, 87·5)	10·4	(9·4, 11·6)
1995	1·3	(1·0, 1·8)	35·2	(30, 41·4)	5·3	(4·6, 6·1)
1996	2·3	(1·9, 2·9)	132·8	(122·3, 144·3)	17·7	(16·4, 19·2)
1997	1·8	(1·4, 2·4)	68·8	(61·4, 77·2)	9·8	(8·8, 10·8)
1998	1·4	(1·0, 1·9)	79·7	(71·7, 88·6)	10·7	(9·7, 11·8)
1999	2·1	(1·7, 2·7)	109·3	(100, 119·6)	15·0	(13·8, 16·3)
2000	0·6	(0·4, 1·0)	29·5	(24·8, 35·0)	4·1	(3·5, 4·8)
2001	1·6	(1·2, 2·1)	68·9	(61·6, 77·0)	9·7	(8·8, 10·8)
2002	1·7	(1·3, 2·2)	84·8	(76·8, 93·7)	11·8	(10·8, 13)
2003	2·8	(2·3, 3·5)	171·0	(159·6, 183·3)	23·3	(21·8, 24·9)
2004	2·3	(1·9, 2·9)	131·0	(121·1, 141·7)	18·1	(16·8, 19·5)
2005	1·6	(1·2, 2·0)	30·0	(25·5, 35·3)	5·1	(4·4, 5·8)
2006	1·9	(1·5, 2·4)	115·3	(106·3, 125·2)	15·9	(14·7, 17·2)
2007	1·7	(1·3, 2·2)	89·8	(81·9, 98·5)	12·6	(11·6, 13·8)
2008	2·1	(1·6, 2·6)	71·3	(64·4, 79·0)	10·7	(9·8, 11·8)
Mean (1990–2008)	1·6	(1·3, 1·9)	76·6	(56·6, 96·7)	10·6	(7·9, 13·3)

Our mean estimates of influenza-associated deaths are 13 or 17 times higher than the number explicitly recorded as due to influenza in the Mortality Collection (mean of 23·4 deaths per annum; range: 2–95; see Table 4).

**Table 4:** Comparison of observed influenza deaths (those recorded in the Mortality Collection) (1990–2008) with Poisson model estimates for respiratory and circulatory and all medical cause data, for all ages

Year	Observed influenza deaths	Estimated influenza-associated deaths (respiratory and circulatory)	Estimated influenza-associated deaths (all medical causes)
1990	46	207·5	252·1
1991	25	40·2	31·2
1992	18	173·2	218·3
1993	28	200·7	239·2
1994	43	286·4	367·2
1995	30	184·8	187·7
1996	95	491·9	641·6
1997	15	305·8	355·4
1998	7	292·3	393·2
1999	27	418·4	552·8
2000	2	114·8	152·2
2001	9	283·3	363·3
2002	9	331·6	449·0
2003	12	637·2	897·6
2004	28	498·5	707·3
2005	14	207·1	201·7
2006	17	431·6	639·5
2007	10	356·1	515·4
2008	9	348·4	444·0
Mean (1990–2008)	23·4	305·8	400·5

**Figure 2:**
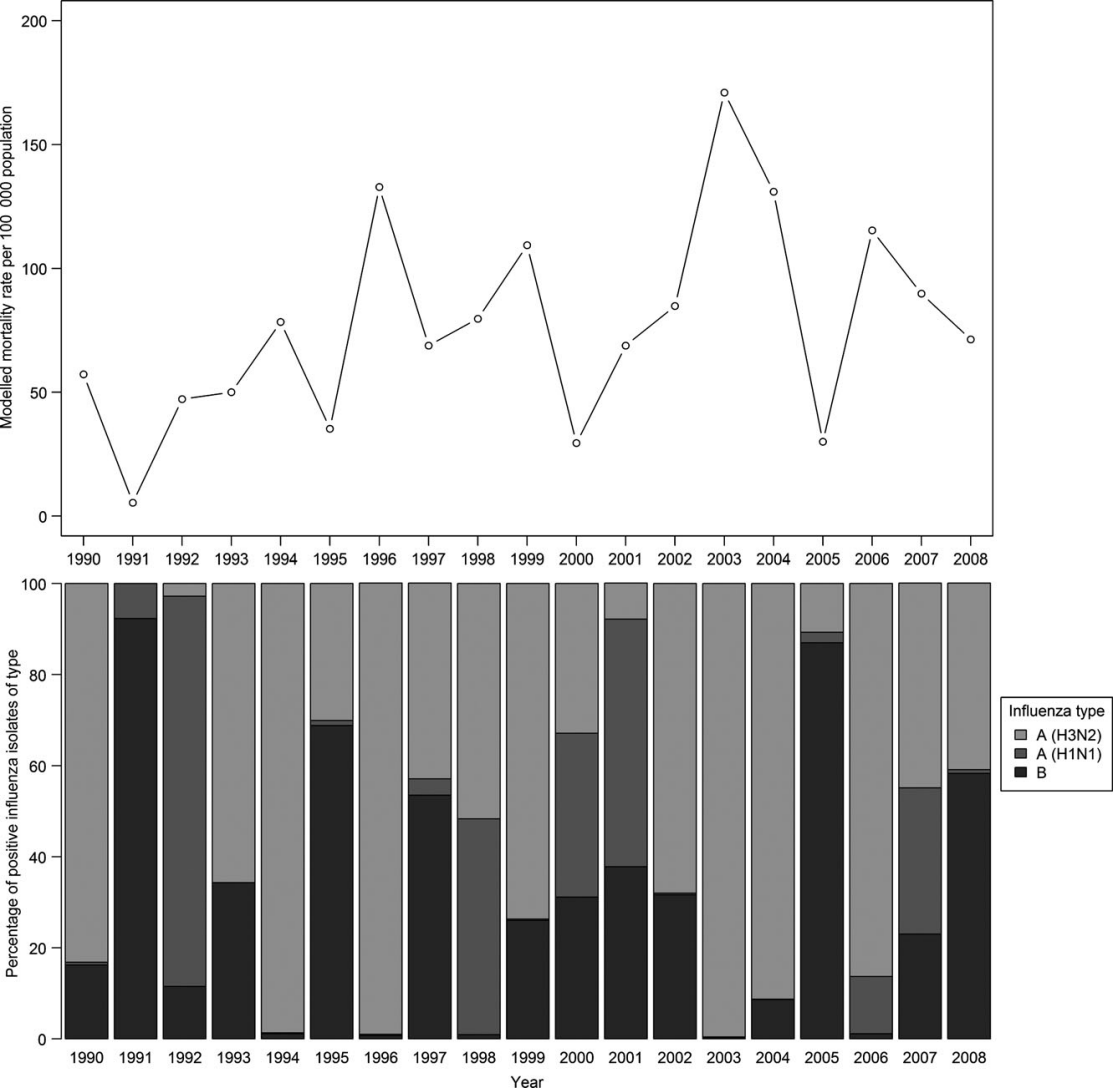
Poisson regression model estimates of influenza-associated mortality rates in those 65 years and over (based on all medical causes data); percentages of total identified influenza isolates by type and subtype, 1990–2008 (source: ESR).

Influenza mortality was highest in years dominated by influenza A(H3N2) compared with influenza A(H1N1) and influenza B (Figure 2).

## Discussion

This study estimates an average of 306–401 influenza-associated deaths per year in New Zealand (the lower estimate from modelled respiratory and circulatory deaths; the upper estimate from all medical deaths). Influenza was associated with 2·2% of respiratory and circulatory deaths and 1·6% of all medical deaths. As expected, the majority of influenza-associated deaths were in those aged 65 plus.

Annual modelled estimates varied considerably, from 31 influenza-associated deaths in 1991 to 898 in 2003. The variability of the impact of seasonal influenza appeared to be influenced by the dominant circulating influenza virus. These estimates also demonstrate the limitations of influenza diagnosis, with an average of only 23 influenza deaths per year recorded in the Mortality Collection.

This study's estimates are broadly comparable with previous estimates for New Zealand and other developed countries. The average estimate (all medical cause data) is lower than the previous New Zealand study estimate of around 490 deaths per year.^4^ For those aged 65 plus in New Zealand, the average rate of 61·0 influenza-associated deaths per 100 000 persons (respiratory and circulatory data analysis) is similar to the corresponding estimate of 66·1 deaths per 100 000 in the USA.^8^

Poisson modelling enabled the use of robust influenza circulation data in mortality estimates. We reduced the influence of circulating RSV on our estimates, yielding a better model fit (under 65: no RSV AIC = 7512; RSV model AIC = 7362; aged 65+, no RSV model AIC = 9913; RSV model = 9318). The Poisson model is limited by an inability to account for interconnectedness between influenza deaths, such as during seasonal epidemics; use of a log-link has been criticised as inappropriate for influenza modelling, although results appear similar to using a linear-link.^9^

As influenza A specimen subtyping was incomplete, we cannot account for different effects of H1N1 and H3N2 on mortality. Furthermore, total numbers of tested specimens were not available as a denominator, so we cannot fully account for influences of increased testing rates over time. Positive specimen counts will also be influenced by commencement of sentinel surveillance in 1991 and diagnostic introduction of rapid antigen testing and PCR during the study period. However, specimen counts have been used previously in Poisson modelling, where mortality patterns were similar by age and time to Serfling estimates.^10^

It would be valuable to apply the Poisson model to hospitalisation data to estimate the serious morbidity caused by influenza. The larger number of hospitalisation events would allow us to estimate inequalities by ethnicity and other sociodemographic factors. There are significant health disparities in New Zealand; Māori and Pacific peoples have higher rates of hospitalisation for infectious diseases,^11^ including both seasonal^12^ and pandemic^13^ influenza.

These results show the large public health impact of influenza and the variability in mortality between years. Our estimates do not indicate a clear decline in influenza-associated mortality over this period.

In summary, this study demonstrates the continued impact of influenza on population health in New Zealand: this evidence will better equip public health policy makers to review current influenza strategies and to identify additional actions to effectively tackle this persistent and important global health concern.

## References

[1] Warren-Gash C, Smeeth L, Hayward AC (2009). Influenza as a trigger for acute myocardial infarction or death from cardiovascular disease: a systematic review. Lancet Infect Dis.

[2] Mills CF, Tobias M, Baker M (2002). A re-appraisal of the burden of infectious disease in New Zealand: aggregate estimates of morbidity and mortality. NZ Med J.

[3] Thompson WW, Weintraub E, Dhankhar P (2009). Estimates of US influenza-associated deaths made using four different methods. Influenza Other Respir Viruses.

[4] Public Health Commission (1996). Influenza: The Public Health Commission's Advice to the Minister of Health 1995–1996.

[5] Ministry of Health (2011). Immunisation Handbook 2011.

[6] Thompson WW, Shay DK, Weintraub E (2003). Mortality associated with influenza and respiratory syncytial virus in the United States. JAMA.

[7] Clayton D, Hills M (1993). Statistical Models in Epidemiology.

[8] Centers for Disease Control and Prevention (2010). Estimates of deaths associated with seasonal influenza - United States, 1976-2007. MMWR Morb Mortal Wkly Rep.

[9] Thompson WW, Shay DK, Weintraub E (2003). Estimating deaths due to influenza and respiratory syncytial virus—reply. JAMA.

[10] Newall A, Viboud C, Wood J (2010). Influenza-attributable mortality in Australians aged more than 50 years: a comparison of different modelling approaches. Epidemiol Infect.

[11] Baker MG, Telfar Barnard L, Kvalsvig A (2012). Increasing incidence of serious infectious diseases and inequalities in New Zealand: a national epidemiological study. Lancet.

[12] Jennings L, Huang QS, Baker M (2001). Influenza surveillance and immunisation in New Zealand, 1990–1999. N Z Pub Health Rep.

[13] Baker MG, Wilson N, Huang QS (2009). Pandemic Influenza A (H1N1)v in New Zealand: the experience from April to August 2009. Euro Surveill.

